# Status of Air Pollution in Botswana and Significance to Air Quality and Human Health

**DOI:** 10.5696/2156-9614-7.15.8

**Published:** 2017-09-07

**Authors:** Modise Wiston

**Affiliations:** Department of Physics, University of Botswana, Private Bag 0022, Gaborone, Botswana

**Keywords:** air pollution, air quality, atmosphere, exposure, human health

## Abstract

**Background.:**

Air pollution is an important issue in developed and industrialized countries. The most common sources of air pollution are anthropogenic activities such as construction dust, vehicular emissions and mining. For low- and middle-income countries, biomass burning and indoor heating are the leading sources of air pollution. As more of the world undergoes development and human populations increase, industrialization is also increasing, along with the potential for air pollution.

**Objectives.:**

This article reviews the status of air pollution to raise awareness of air quality and human health in Botswana.

**Discussion.:**

Since independence, Botswana has experienced one of the highest economic development growth rates in the world. These changes have occurred as a result of economic growth and resource utilization associated with increased industrialization. However, there is growing worldwide concern about the effect and impact of pollution due to industrial growth. Botswana is ranked amongst the most polluted countries with serious air pollution, despite a population of just over 2 million.

**Conclusions.:**

Rapid development and increased urbanization have had a major environmental impact around the world. This increased growth has the potential to lead to air quality degradation. Significant health threats are posed by industrial and vehicular emissions, especially in urban and peri-urban areas where the population is most concentrated. It is important that the linkage between air pollution and health effects is fully examined across all scales of life, especially in developing countries. In addition, programs should be devised to educate the public about the pollution impacts on health.

## Introduction

For many decades, air pollution has been associated with the export of pollutants from urban (large cities and industrial areas) to rural and other distant areas. The perception has been that only industrialized or developed countries are susceptible to air pollution. However, this is no longer the case as even less developed and middle-income countries experience high particle densities and significant air pollution.^1^ Air pollution has a significant effect on air quality due to its wide-ranging potential consequences to human health, ecosystems, visibility, weather modification, radiative forcing and changes in tropospheric chemistry.^2^ Pollutants occur in the form of gases and particulates from biological/natural and anthropogenic (human-made) processes released into the atmosphere. Air pollution is pervasive across Africa; the continent is quickly urbanizing and pollution from vehicle exhaust, wood burning, dusty dirt roads, power plants and other industrial activities has reached high levels in many cities. In some of the most populous cities and/or national capitals, pollutant concentrations exceed threshold limits (i.e. levels considered safe by the World Health Organization).^3^,^4^ As a result, populations in these centers are likely to be at risk of air pollution problems. Indoor air pollution caused by cooking with wood and other sooty fuels such as charcoal and cow dung is also an issue of concern. Migration from the countryside to urban areas increases emissions and exposure to pollutants.^5^

Although there is no national record of air quality problems, many potentially hazardous air pollution conditions exist, especially in areas most impacted by frequent biomass burning, and industrial emissions.^6^ For example, in the southern part of Africa, winter is normally dry and characterised by low temperatures, veld fires (which sometimes start accidentally), human-induced biomass burning, coal burning and other industrial operations for energy production, all of which raise pollution levels. Moreover, biomass burning is a common activity in southern Africa and is not controlled or regulated.

### Air Pollution in Low- and Middle-Income Countries

Pollution has a significant effect on the environment and its proper management is crucial to mitigate the effects of its release. Continuous burning activities (e.g. farming practices, human settlements and energy sources) add to the regional pollutant burden as evidenced by thick smoke, especially in winter.^7^,^8^ Traces of anthropogenic emissions are also visible from urban/industrial sources. Emissions from vehicles are predominant in densely populated cities/towns, and visible in the morning and afternoon during peak hours. For example, the transport sector has expanded rapidly in recent years, resulting in doubled car fleets over the last few decades in countries such as Botswana and Zimbabwe.^9^,^10^ Equally visible are smoke emissions from industrial and indoor heating from urban areas and other homesteads. However, the greatest threat of indoor air pollution occurs in settlements (both rural and urban), as many people continue to rely on traditional fuels for cooking and heating.^11^ Studies have also indicated that high air pollution levels have substantial effects on human health and infant mortality rates.^12^,^13^,^14^,^15^ As particulates (e.g. dust, smoke, vehicular emissions and other small suspensions) are released into air, they negatively impact air quality by polluting the air.

**Abbreviations**
*DWMPC*: Department of Waste Management and Pollution Control
*NO_X_*: Nitrogen oxides
*PM*: Particulate matter
*SADC*: Southern African Development Committee
*SO_2_*: Sulphur dioxide
*WHO*: World Health Organization

Not all of the pollution disposed into the air is neutralized quickly; some pollutants can remain for longer periods before being removed. This depends upon factors such as the flux or concentration of pollutants released, their lifetimes, meteorological parameters prevailing at the time (e.g. wind, precipitation) as well as atmospheric processes (e.g. chemical reactions and stability). By-products of chemical reactions are discharged into the atmosphere, altering its chemical composition and spreading fine particulates; some of which, if inhaled, can attack the lung tissues, and may cause respiratory problems and/or death. Acute respiratory infections are among the leading causes of diseases worldwide and have been linked with exposure to pollutants from domestic biomass fuels in developing countries.^16^ These infections are also one of the leading causes of child mortality, with most fatalities among children under five years of age in developing countries.^17^,^18^ Air pollution is linked to a number of human health and environmental impacts (e.g. respiratory diseases, heavy metal poisoning) and affects lakes by increasing acidic levels or nutrients that affect water quality and aquatic life. Polluted air can also cause shorter lifespans and pose other health hazards to human health.^19^

Air quality assessments are carried out to determine whether threshold limits for particular pollutants are being exceeded. A threshold limit quantifies the maximum average concentration of contaminants to which people may be exposed to in a given time without injury to their health.^20^ Air quality guidelines and set standards are fundamental to effective pollution management and provide the link between the source of emissions (emitter) and the user/target (recipient). Data is collected from major polluting sources, and an emission inventory provides a current and comprehensive understanding of air pollution emissions within a specific area over a specified period of time.

While economic growth is important to any country's advancement, worldwide experience with growth highlights concern about the side effects and impacts of pollution into the atmosphere. Improper disposal of pollution has become one of the hindrances to development, especially due to a lack of understanding of the consequences and/or lack of policies and regulations. This is a common phenomenon in developing countries in their pre-industrial stage and for countries on the verge of industrialization, and has been an ongoing issue in Botswana (an upper middle-income country). Waste management, pollution and poor urban conditions are some of the major challenges associated with development in Africa. The purpose of this paper is to give an overview of the state of local pollution and highlight some of the specifics of air pollution issues in Botswana. Special attention is given to the concentrations and impacts of chemical pollutants on air quality. There has been relatively little research on regional air quality and pollution impacts in Botswana.

## Discussion

**Figure 1:**
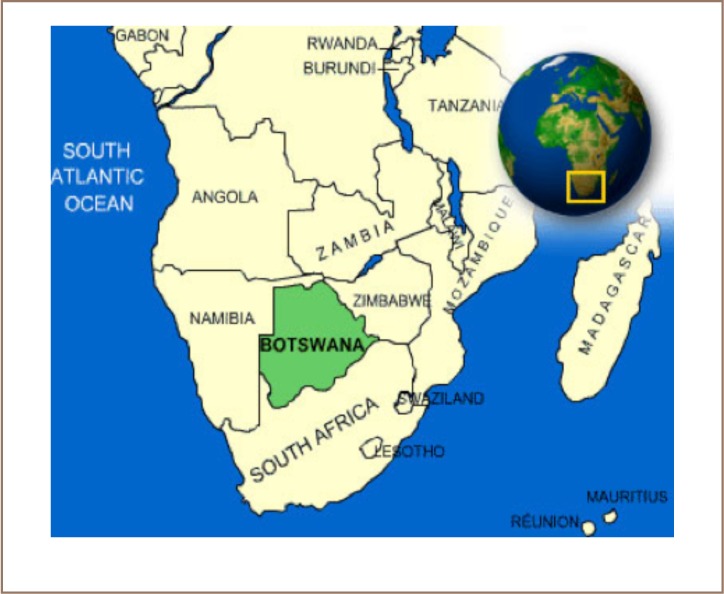
Location of Botswana within southern Africa

Botswana is a landlocked country, located in the center of southern Africa (*Figure 1*), with an population expected to increase from about 2,024,904 in 2011 to about 2,565,855 in 2026.^21^ Most of the urban areas and modern developments are located along the eastern part of the country, and much of the population is concentrated in the east, where there is sufficient space for agriculture, better road networks and other modern facilities. Industrialization has been highly encouraged since independence and continues to be an essential component of developmental efforts. Industrialization is regarded as a ‘key engine’ to economic growth and prosperity, and is the main driver of economic growth. These changes will continue as a result of economic gain and lead to increased resource utilization and consumption.^22^ Botswana is reported to be one the most highly polluted countries, with high emissions and serious air pollution,^23^ despite its low population. For example, about 40% of an estimated 330 child deaths due to acute lower respiratory infections are attributable to household air pollution.^24^

Botswana relies on different sectors (mining, tourism, agriculture and manufacturing) for economic development. Mining has been the leading contributor to the gross domestic product (GDP) since independence.^25^,^26^ While other sectors (textile, industry and manufacturing) also play a role, they are smaller scale operations and the import rate is greater than the export rate for most goods. For example, more than 50% of the country's electrical supply is imported from other countries, especially neighboring South Africa. As much as these sectors contribute substantially to development, they also have a direct impact on pollution in the country and the region, although this problem does not arise solely from industrial air pollutants. There is also a significant amount of pollution coming from other sources such as fires and other common natural sources, such as wind-blown dust.

### Pollution Policy and Regulation in Botswana

Botswana has experienced one of the highest economic development growth rates since independence and is facing a myriad of environmental problems due to its rapid development. Examples include increased solid waste generation, as well as air pollutants from various sources (e.g. mining, fuel burning and vehicular emissions).^11^,^27^ Different types of pollutants require specific source point treatments and disposal methods to reduce negative impacts on the environment. Ambient air quality is monitored in accordance with the Atmospheric Pollution Prevention Act (APA) that calls for the ‘prevention of atmospheric pollution by industrial processes in declared controlled areas’.^28^ The government of Botswana has factored environmental sustainability into the national agenda, one major milestone being the establishment of Environmental Impact Assessment (EIA) legislation in 2005, which requires all new developments to be assessed for their environmental impacts.^29^

Botswana also created a policy for the safeguard of air quality—the main goal being to have a modern air pollution monitoring and surveillance system to assist in planning and other decision-making processes. Some of the objectives include:

- establishing a sound scientific basis for policy development
- assessing population and ecosystem exposure to pollution
- establishing a database for public information and awareness
- identifying pollution sources and risks
- evaluating long-term trends
- establishing a basis for abatement strategy planning.^28^,^30^

For all operations with the potential to pollute, specific policies, acts/regulations and recommendations should be met in order to ensure adherence to air quality standards (*Table 1*). Such operations should be regularly assessed to ensure the safety of employees, the environment and industry sustainability. Some of the acts/regulations under which polluting processes operate include, but are not limited to: the APA of 1971; Botswana Strategy for Waste Management of 1978 (addresses how waste management is to be carried out to protect human health and the environment and ensures prudent use of natural resources); Factories Act of 1979 (provides for occupational health and safety conditions in factories); and the EIA of 2005.^28–32^ The EIA defines environmental policies to assess the potential effects of planned developmental activities, and determine and provide mitigation measures to address adverse impacts on the environment. The EIA also ensures that monitoring and evaluation of environmental impacts are put in place during operations.

**Table 1:**
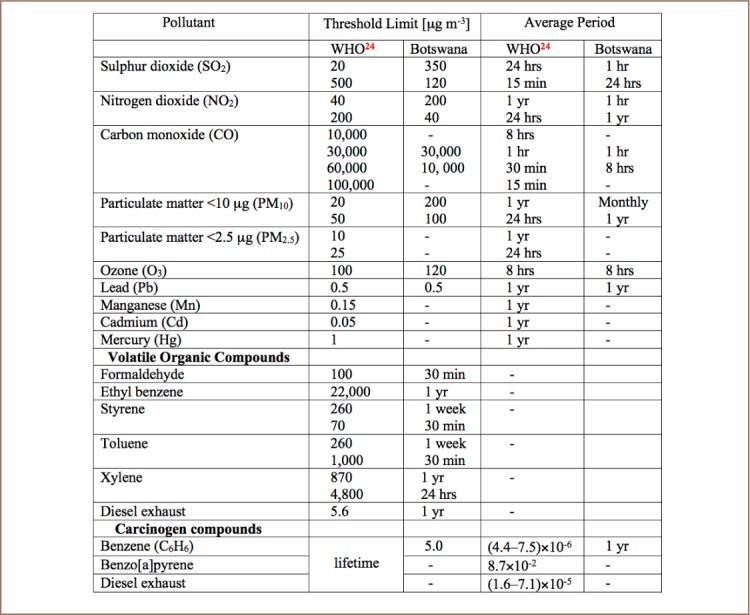
WHO and Botswana Air Quality Guidelines for Common Air Pollutants. (Data adapted from Mmolawa and Keabetwe^34^ [obtained from BOS 498: 2012]^48^ and Schwela)^9^

The Air Pollution Control Division in the Department of Mines aims to ‘promote and ensure sustainable industrial development and improvement of the standard of living in Botswana by controlling pollution at sources to protect the environment, public and health welfare.’ Some of its objectives include: (i) minimizing atmospheric pollution by industries and other anthropogenic activities, (ii) continuous monitoring of air quality within the country to determine exposure levels of air pollution to the public and environment and (iii) promoting the concept and practice of pollution prevention at the source.^28^ The Department of Waste Management and Pollution Control (DWMPC) was also established with the mandate to control air pollution from primary sources, providing management of controlled and hazardous wastes, as well as planning, facilitating and implementation of waste management strategy. The mandate applies to all government institutions and all activities that deal with pollution and waste. One of the goals is to provide for the prevention and minimization of pollution (land, water or air), control and remediation measures. The DWMPC issues a license to businesses and firms operating recycling facilities.^33^ Different exposure levels are defined such that concentrations from different pollutants must fall within set standards, as humans are at high risk when exposed to increased levels or with prolonged exposure. Table 1 highlights some of the air quality thresholds for a number of pollutants in Botswana. Regionally, several agreements have been signed committing the Southern African Development Committee (SADC) member states to improving air quality standards—including the Dakar Declaration to phase out leaded gasoline by 2005 and the Harare Resolution of 1998 on the initiation of the SADC Protocol on Regional Air Quality and Atmospheric Emissions. Furthermore, all SADC member states are parties to various multilateral environmental agreements, including the United Nations Framework Convention on Climate Change, the 1999 Basel Convention and the 1994 Bamako Convention (www.sadc.int).

### Sources of Air Pollution in Botswana

Although Botswana is not highly industrialized, several industries such as metal processing have recently been introduced. Sources of air pollution include industrial operations, manufacturing, smallscale plants, smelters, stone/sand crushers, traffic emissions, waste and household fires. Household burning of fossil fuels (e.g. wood and biomass) remains one of the major energy sources used for cooking, heating and power generation. In addition to these anthropogenic sources, natural sources also contribute a significant amount of pollution, including the Kalahari Desert and natural fire eruptions. One other significant factor is the high rate at which the number of vehicles has increased in Botswana in recent decades. There is a significant amount of vehicle importation, especially of cheap used Japanese vehicles, most of which are not properly maintained after purchase. These vehicles are reconditioned older model cars discarded from industrialized countries and there is a continuing problem of lead additives in petroleum.^10^ There is also a significant number of privately owned vehicles, leading to increased traffic congestion on the road despite the poor state of the road network. The rising number of automobiles leads to high levels of traffic-related pollution.^5^,^34^

A vast amount of mineral dust is also generated from the Kalahari (covering much of the western part of the subcontinent), which has a significant impact on regional pollution and climate. Dust generation results from rapid soil loss and desertification either through industrialization or resource utilization, leading to increased windblown soil erosion, forest fires and deforestation. Fine dust particles can be lifted up to higher altitudes and transported over long distances away from their source regions. Their effects can be felt not only locally, but also in regions far away from their sources.^35^ As a result, many locations have the potential to be affected by particles transported from desert areas.^36^ This effect is attributed to intensified grazing pressure along with climate change and the associated reactivation of the Kalahari dune field after a millennia of inactivity.^37^,^38^,^39^ Previous studies have reported that overgrazing is one of the major contributors to vegetative loss over the Kalahari.^40^ They reported an increase in the grazed area in the Kgalagadi district in southwest Botswana from 13,000 to 32,000 km^2^ between 1950 and the 1990s. Climate and land use changes in the southern Kalahari encourage dust emission, and dust generation involves a balance between the area becoming susceptible to dust emission and depleted of its fine sediment supply.^41^,^42^ Some of the major pollutant sources in addition to those highlighted above include power plants, mines and industries in the major cities and towns in Botswana.

The Bamangwato Consolidated Limited (BCL) copper-nickel mine in Selebi Phikwe has been a major source of pollutants. Due to the town's growing population, the mine and its smelter plant are now in close proximity to residential sites.^25^ Sources of air pollution have been compounded by the release of sulphur dioxide (SO_2_) and other toxic gases from mining activities, resulting in smoke released into the atmosphere, and respiratory problems among the town residents.^43^ Very often, the air around the town and surrounding areas would be clouded with thick smoke—sometimes lasting for more than 24 hours. Prior to its closure in 2016, emissions occurred at all stages, including tailing piles, crushed and waste rocks/sand and gaseous species from the smelter.

Morupule Power Station, located about 6 km west of Palapye is another major potential source of pollution. This power plant generates electricity using pulverized coal mined at the Morupule Colliery (Ltd), just adjacent to the station. The coal burns and reacts with oxygen to produce gaseous pollutants such as carbon dioxide, carbon monoxide, SO_2_ and nitrogen oxides (NO_x_ [where NO_x_ = nitric oxide + nitrogen dioxide]), which are then released into the atmosphere through tall chimneys.^44^ There are two plants, Morupule A and B, in operation next to each other. Due to the increasing demand for electricity and the country's effort to cut outside electrical supply costs, the power station has been upgraded. Electricity has mainly been imported from Eskom (South Africa) with a peak demand of 434 MW satisfied through internal generation and imports.^44^ The second plant was constructed to assist with load shedding that has been a concern in recent years.^45^ Once fully operational, Morupule B is expected to produce a total of about 600 MW of electricity (only about 132 MW is generated from Morupule A), with an annual requirement of about 3 million tons of coal supply.^46^ Botswana's energy demand was estimated at about 3660 GWh in 2008 (peak load of 500 MW), and is projected to grow at about 6% per annum, reaching 5300 GWh in 2017.^45^

Although Botswana has not established emissions standards for power stations, the Air Pollution Prevention Act of 1971 requires the application of best practices to control emissions from the site.^44^ The Botswana Power Corporation aims to undertake an environmental audit for the operation as well as annual air quality monitoring to address the problem. Necessary measures are to be implemented to ensure that operations from both plants do not exceed the air quality standards for Botswana or the World Bank.^44^ In addition, there is another small coal-fired plant in Makoro manufacturing face bricks from clay. Production has now grown over the years and supplies bricks to almost all parts of the country. Clay is burnt with charcoal (about 200 tons monthly) from the Morupule Colliery mine. However, there is no monitoring of pollutants at or around the plant. According to the plant authorities, all the products are disposed of through chimneys from the plant into the atmosphere.

There are several stone and sand crushing operations around the country, crushing stones into different particle sizes, from concrete to fine sand. Large quantities of crushed soil are normally piled in open spaces and easily blown away by winds in all directions. Most of the sites are not fenced, other than the surrounding vegetation that acts as ‘source sinks’ to the wind-blown dust. The only form of dust control is suppression from the crushing machine and conveyor belts. One example is the Nata-Phikwe quarry, just adjacent to the coppernickel mine in Selebi Phikwe. Dust generated from these operations poses a potential health hazard, particularly respiratory problems from inhaled pollution. These respiratory-related tract problems are linked to effects of air pollution from the mining and smelting activities.^47^

Another coal-fired power station (located in Mookane, about 100 km south east of Mahalapye) is proposed and is expected to be one of the largest power plants in the country. Botswana has considerable coal deposits—one of the largest potential reserves untapped in the world at over 212 billion tons.^46^ There are currently four commercially significant coal deposits (Morupule, Mmamabula, Sese and Mmamantswe), with an envisaged export industry of at least 36–90 Mt/a. It is estimated that as much as two-thirds of Africa's coal resources are found in Botswana. Similarly, several other mining sites exist around the country (e.g. diamond explorations), which also generate pollutants, some very close to townships.

In addition to the sites described above, some cities and major towns around the country also make a substantial contribution to local air pollution. For example, the largest and national capital city (both administrative and industrial) in Botswana is Gaborone, located to the southeast. Main pollution sources include fuel used in various industries (e.g. brewing, soap manufacturing, textile, cement bagging, pipe manufacturing) and small-scale chemical and other industries.^30^ The biggest pollution source is gas-produced steam from the coal-operated factories, while non-industrial sources include government institutions (e.g. schools, hospitals, restaurants and mobile food stalls) using coal/gas energy sources and open burning of waste and firewood, particularly during the dry and cold winter. Despite the technological advancement and growth rate of the population, raw materials are still used by large communities as sources of energy. Residents often burn heavy, polluting fuel, including firewood and cow dung for heat and energy that can produce thick smoke. In addition, the increasing traffic congestion in Botswana, particularly in Gaborone, contributes significantly to air pollution. The main market for imported vehicles is in Mogoditshane (just adjacent to Gaborone in the west), where almost all the vehicle imports are sold or distributed. Consequently, Gaborone is one of the most polluted world cities in terms of air quality rating according to 2000–2005 global ambient air pollution concentrations and trends, even though the country is only averagely developed and is about 80% desert.^48^

The second largest city is Francistown, located in the northeast and close to the country's border with neighboring Zimbabwe. There are several industrial and manufacturing operations in Francistown, including Botswana Meat Commission, mining and food processors. Other sources of pollution include a sorghum beer brewery (operated by Botswana Breweries), and small-scale industries. Coal-fired boilers, open burning and traffic emissions also significantly contribute to atmospheric pollution. Adjacent to the city is the Tati copper-nickel mine which employs many local people, and the city is surrounded by other villages. Located further south of Gaborone is Lobatse, one of the fastest growing industrial towns with several industries and factories. Major sources of pollution include the Botswana Meat Commission (the largest meat processing industry in Botswana) and the Lobatse Clay works. Smallscale sources include firewood from cooking, open burning of refuse and traffic, which contribute to atmospheric pollution in and around the town.

### Air Quality Monitoring in Botswana

Air quality management is an important tool for assessing the status of air pollution in order to design and implement standards for the control and measurement of air pollution, and to meet air quality objectives. Air pollution mitigation strategies are designed to identify pollution problems and provide solutions at the town, regional, national and international level. Ambient monitoring is concerned with on-site measurements of major emissions (e.g. particulate matter (PM), NO_x_, SO_2_ and carbon monoxide).

Routine air pollution monitoring in Botswana began in the mid-1970s, with monitoring of SO_2_ gas from three stations only.^49^,^50^ The number of monitoring stations increased in 1995 and expanded to include pollutants such as NO_x_ and other substances, and ground level ozone monitoring began in 1996. The majority of SO_2_ emissions have come from the copper-nickel smelter at Selebi Phikwe, while major towns with high traffic volume show significant elevation of NO_x_ concentrations during peak hours. Currently, there are several air pollution monitoring stations across Botswana, operating under the DWMPC. Several studies undertaken on the impacts of smelter emissions showed serious impacts on both vegetation and population in Selebi Phikwe and surrounding villages, although these studies did not quantify the extent of the impacts.^25^,^40^,^51^ Measured concentrations of SO_2_, nitrogen dioxide and ozone indicated potential impacts of air pollution on vegetation and human health, with the SO_2_ guideline exceeded in Selebi Phikwe.^49^,^52^ Other studies conducted on particle concentrations in Gaborone resulting from biomass burning indicated an increase in particle concentrations of up to 1700 cm^−3^ and 1894 cm^−3^ during the peak time of winter.^27^,^53^ Another study conducted on indoor air pollution from household fuels in Gaborone from winter to spring (July–September 2007) showed that people from lowincome groups reported more health effects than those in medium and high-income groups.^53^ Although the study was conducted in one part of the city, particle concentrations were increased due to the burning of raw materials such as cow dung, wood, plastic and Chibuku (local beer) cartons for cooking and heating of homes.

Mean PM concentration indexes estimated for populations around the world reported a PM_2.5_ air pollution population exposure level (% of total) of 45.44 in Botswana in 2013, exceeding the annual average World Health Organization (WHO) guideline.^9^,^11^,^54^ This index expresses the percentage population exposed to ambient PM_2.5_ concentrations exceeding the WHO guideline value, given as the portion of a country's population living in places where the mean annual concentration of PM_2.5_ is greater than 10 μg m^−3^. Overall, these ambient concentrations could mask an individual's true exposure, which may vary with an individual's proximity to the polluting sources during periods when in use/operation or at work. Table 1 shows threshold limits for some common air pollutants in Botswana and globally (WHO guidelines).^24^ Data are shown for some of the major regulated pollutant species that are regularly monitored to assess pollution levels.

### Challenges and Limitations to Air Quality Monitoring in Botswana

As highlighted above, there are a number of challenges with regard to air quality monitoring, data collection and information dissemination in Botswana, as in most developing countries. Some of these challenges include lack of regular site visitation and monitoring programs. There remains a lack of transparency in various aspects of air pollution control, poor environmental regulations or weak institutional mechanisms to enforce regulation ranging from effluent disposal through control and monitoring strategies. In addition, regulatory operators do not always adhere to existing air quality rules and regulations. While the DWMPC is the main body to oversee, facilitate and implement the management and control of air pollution, assessment must depend on industries (polluting sources) and/or individuals for data without regular visitation and site assessments. This can negatively affect the monitoring of pollutants, especially where the programs/instruments are inadequate, posing a risk to the workforce.

If pollution in Botswana is not regulated to specified levels, it can lead to harmful effects. For example, pollution may have long lasting effects on general health and well-being; early exposure in childhood may harm lung development later in life.^55^ The workforce and communities near polluting sources may not be adequately educated about pollution impacts or the use of hazardous materials (e.g. cooking/heating fuels). Similarly, most air quality monitoring systems do not fully address population exposure to toxic pollutants and monitoring is needed to determine if companies meet their operational requirements.^33^ In addition to these challenges, further studies are needed on air quality and pollutant concentrations, especially around potentially polluted places and regular check-up of monitoring stations. There are also a few limitations to the present study. No measurement data was collected or obtained from the monitoring stations in the present study which could be compared with real world measurements. This highlights a major concern in Botswana as data is not readily available to researchers or the public.

## Conclusions

The combination of increasing migration, motorization and uncontrolled urban growth contributes to the intensification of air pollution, and has led to a concerning increase in pollutant gases and particles. Like many developing nations, Botswana has experienced a significant amount of pollution due to rapid growth in its urban population, industrialization and rising demands for energy and motor vehicles.^47^ The combined effect of population dynamics and economic development has a noticeable effect on the environment in terms of increased waste generation and poor waste management.

Major issues that need to be addressed include the extent and impact of air pollution, development of future problems, and required responses. For example, under the Kyoto protocol framework agreement, industrialized countries are obliged to cut their greenhouse gas emissions.^56^ In addition, air pollution is not adequately considered in the planning and placement of pollution sources and residential sites; there is a lack of mitigation measures and/or non-operational emission controls. Occupational safety and health policies should be put in place and/or enforced to educate the general public about the impacts of pollution on health. Workers should limit close contact with pollutants where risky exposure to contaminants is high, receive regular medical checkups, and effective occupational exposure programs and environmental control measures should be implemented. The linkage between air pollution and health effects needs to be fully developed, especially in developing countries. Strong legislation would go a long way towards improving the safety of the workforce and communities. Policy makers (government and other stake holders) need to advocate for pollution impact education and implement measures to mitigate the many adverse effects of air pollution.
